# Time to workforce exit after a Parkinson’s disease diagnosis

**DOI:** 10.1038/s41531-023-00513-0

**Published:** 2023-05-08

**Authors:** Jonathan Timpka, Örjan Dahlström, Maria H. Nilsson, Susanne Iwarsson, Per Odin

**Affiliations:** 1 Division of Neurology, Department of Clinical Sciences Lund, Lund University, Lund, Sweden; 2 Department of Neurology, Skåne University Hospital, Lund, Sweden; 3 Department of Behavioural Sciences and Learning, Linköping University, Linköping, Sweden; 4 Athletics Research Center, Linköping University, Linköping, Sweden; 5 Department of Health Sciences, Lund University, Lund, Sweden; 6 Memory Clinic, Skåne University Hospital, Malmö, Sweden

**Keywords:** Parkinson's disease, Health policy

## Abstract

The impact of Parkinson’s disease (PD) on workforce participation has received little attention even though demographic, lifestyle, and political changes together will result in an increased burden of PD on the working-age population. In this study, we investigate workforce survival after a PD diagnosis, as well as what demographic factors that are associated with workforce survival. As an exploratory outcome, we investigate workforce survival in persons with and without device-aided treatment (DAT). This is a nested case-cohort study based on Swedish national data from 2001–2016. Controls were matched on year of birth, sex, and municipality of residence. The used registers contain data on demographics, social insurance, in- and outpatient visits, filled drug prescriptions, and cause of death on the person-level. A total of 4781 persons with PD and 23,905 controls were included. The median survival until all-cause workforce exit was 43 months among persons that were workforce-active at the time of PD diagnosis, compared to 66 months in non-PD controls. Being female, ≥50 years old at diagnosis, or having a lower education were contributing factors to health-related workforce exit. Persons receiving DAT during follow-up exhibited shorter workforce survival than controls. However, this needs further investigation, particularly as patients have generally already left the workforce at the time for start of DAT. It is evident that PD has grave negative effects on workforce participation. Thus, supportive measures need to start at an early stage after diagnosis, and the development of new interventions is urgently needed.

## Introduction

The effects of Parkinson’s disease (PD) on working ability have received unjustifiably little attention^1^. Just as extending working life participation beyond one’s capacity leads to dissatisfaction, so does forced workforce exit^2^. Improved general health predisposes for later retirement at the population level^3^, which paradoxically increases the risk of interference of PD on the ability to work^4,5^. It is evident from PD-related research on workforce participation that not only worsened manual dexterity, but also anxiety, sleeping difficulties, and difficulties eating or drinking are factors that contribute to leaving work early^6^. The results point to fatigue^7^, rigidity/bradykinesia, and stress intolerance as particularly detrimental to the working ability^2^. Together, this complex constellation of symptoms contribute to the fact that among persons with PD, employment has historically been rare beyond 10 years after diagnosis^1^.

Previous studies on workforce participation and PD have mainly included small samples and used questionnaire data^1,8,9^. We wanted to employ large-scale real-world data to chart workforce participation after a PD diagnosis in the 21st century. The infrastructure for register studies is strong in the Nordic countries. Although this infrastructure has been used to show that the sickness absence and healthcare spending is increased already prior to a PD diagnosis^10,11^, its potential is under-utilized in relation to the scarce knowledge concerning working ability and PD.

Evidence of beneficial effects on workforce participation is an important justification of potential new therapeutic options for PD, such as stem cell therapy^12^. Exploratory studies on the current generation of device-aided treatment (deep brain stimulation, levodopa-carbidopa intestinal gel, or subcutaneous apomorphine infusion; DAT) indicate that there are positive effects on the working ability of persons with PD and motor complications^13–15^.

The primary objective of our study was to determine the time to workforce exit in persons diagnosed with PD in the total Swedish population. The second objective was to explore what factors that are associated with workforce exit in PD patients. A third aim was to investigate how DAT affects workforce participation in PD.

## Results

### Cases and controls

A total of 4,781 cases diagnosed with PD and 23,905 controls were identified from the base population during the ten-year inclusion period (Table 1). A majority of persons included were male (59%) and the median age was 59 years. PD cases were less likely to be workforce active at the time of diagnosis than matched controls (PD, control, *χ*^2^ test, [64.3%, 73.9%, *P* < 0.001]), less likely to emigrate during the follow-up (0.4%, 0.8%, *P* = 0.001), more likely to die during the follow-up (20.5%, 7.6%, *P* < 0.001), and had slightly longer education (*P* < 0.001).

**Table 1:** Descriptive information on the study population.

	PD	Controls	*P*^a^
*n*	4781	23,905	
Age (years), m (Q1–Q3)	59 (54–62)	59 (54–62)	1.000
Sex, male/female,%	62.6/37.4	62.6/37.4	1.000
Place of birth, *n* (%)			0.736
Domestically w/ both parents born domestically	3957 (82.8)	19,728 (82.5)	
Domestically w/ one parent born domestically, one parent born abroad	211 (4.4)	1062 (4.4)	
Domestically w/ both parents born abroad	36 (0.8)	269 (1.1)	
Abroad	577 (12.1)	2846 (11.9)	
Education^**b**^, *n* (%)			<**0.001**
Primary and lower secondary education, less than 9 years	644 (13.6)	3350 (14.0)	
Primary and lower secondary education, 9 years	507 (10.7)	2679 (11.2)	
Upper secondary education	2072 (43.8)	10,881 (45.5)	
Post-secondary education, less than 2 years	220 (4.7)	1170 (4.9)	
Post-secondary education, 2 years or longer	1183 (25.0)	5392 (22.6)	
Postgraduate education	77 (1.6)	253 (1.1)	
Medication during follow-up (ATC)^c^, *n* (%)			
Adamantane derivatives (N04BB)	397 (11.1)	10 (<0.1)	<**0.001**
Levodopa and decarboxylase inhibitor (N04BA02)	3215 (89.9)	210 (1.2)	<**0.001**
Levodopa, decarboxylase inhibitor, and COMT inhibitor (NA04BA03)	1331 (37.2)	11 (<0.1)	<**0.001**
Dopamine agonists (N04BC)	2763 (77.3)	342 (1.9)	<**0.001**
MAOB inhibitors (N04BD)	1771 (49.5)	16 (<0.1)	<**0.001**
Other dopaminergic agents (N04BX)	885 (24.7)	3 (<0.1)	<**0.001**
Antidepressants (N06A)	2209 (61.8)	4672 (26.1)	<**0.001**
Diazepines etc. (N05AH)	669 (18.7)	351 (2.0)	<**0.001**
Anti-dementia drugs (N06D)	516 (14.4)	128 (0.7)	<**0.001 **
Device-aided treatment during follow-up, *n* (%)			
Levodopa-carbidopa intestinal gel	233 (5.1)	0 (0)	<**0.001**
Subcutaneous apomorphine infusion	193 (4.2)	0 (0)	<**0.001**
Deep brain stimulation	267 (5.6)	8 (0.4)	<**0.001**
Workforce participation, *n* (%)			
Workforce active at time of diagnosis	3074 (64.3)	17,668 (73.9)	<**0.001**
Died during follow-up, *n* (%)	978 (20.5)	1815 (7.6)	<**0.001**
Emigrated during follow-up, *n* (%)	17 (0.4)	180 (0.8)	<**0.001**

^a^Mann-Whitney or *χ*^2^ tests depending on the type of data.

^b^Highest attained education at the start of follow-up according to the Swedish Classification of Education (SUN2000).

^c^One or more issued prescription from the time of establishment of the Swedish Drug Prescription Register in July 2005 to the end of follow-up. PD cases/controls with a diagnosis/matching prior to July 2005 have been excluded. *N* of PD cases: 3576, *N* of controls: 17,880.

*PD* Parkinson’s disease, Q1–Q3 1st to 3rd quartile, *COMT* catechol-O-methyltransferase, *MAOB* monoamine oxidase B, *ATC* anatomical Therapeutic Chemical code.

### Workforce exit

Workforce exit was more common among PD cases than among controls at all time intervals in relation to the PD diagnosis, except in the age group 60 years and older (Table 2). In that age group, there were no significant differences in workforce participation between PD cases and controls beyond five years after diagnosis.

**Table 2:** Time point for workforce exit in relation to a Parkinson’s disease diagnosis in different age groups.

	*N* =		Before diagnosis (%)			Within … from diagnosis (%)														
						1 year			3 years			5 years			7 years			10 years		
Age	PD	Control	PD	Control	*P*	PD	Control	*P*	PD	Control	*P*	PD	Control	*P*	PD	Control	*P*	PD	Control	*P*
19–50	186	913	22.0	9.1	<**0.001**	35.5	11.4	<**0.001**	40.9	13.9	<**0.001**	45.7	16.3	<**0.001**	51.6	17.4	<**0.001**	57.5	19.8	<**0.001**
50–59	601	2976	26.1	15.6	<**0.001**	36.6	20.8	<**0.001**	50.2	26.4	<**0.001**	62.9	35.6	<**0.001**	76.9	55.9	<**0.001**	91.7	80.6	<**0.001**
60–64	640	3174	43.8	30.7	<**0.001**	67.8	52.2	<**0.001**	88.6	78.4	<**0.001**	98.6	97.0	**0.025**	99.8	99.4	0.139	99.8	99.8	0.860
All	1427	7063	33.5	21.5	<**0.001**	50.5	33.7	<**0.001**	66.2	48.2	<**0.001**	76.7	60.7	<**0.001**	83.9	70.5	<**0.001**	90.9	81.4	<**0.001**

The table includes PD cases and controls with a date of diagnosis/matching at least 11 years prior to the last date of follow-up. Cases and controls were matched at the time of diagnosis on year of birth, sex, and municipality of residence. Cases/controls who emigrated during follow-up and all controls to cases who emigrated during follow-up were excluded. Repeated *χ*^2^ tests resulted in *P* < 0.001 at all intervals except in the 60–64 age group with workforce exit ≤5 years (*P* = 0.025), ≤7 years (*P* = 0.139), and ≤10 years (*P* = 0.860) after diagnosis. Workforce exit was defined as the first occurrence of either death, two successive years without information from employer on income, 1 year with ≥274 days of sickness pension, 1 year with full pension; two successive years with ≥274 days of sick pay, rehabilitation, or working injury compensation; or two successive years with ≥274 days of unemployment.

**Fig. 1:**
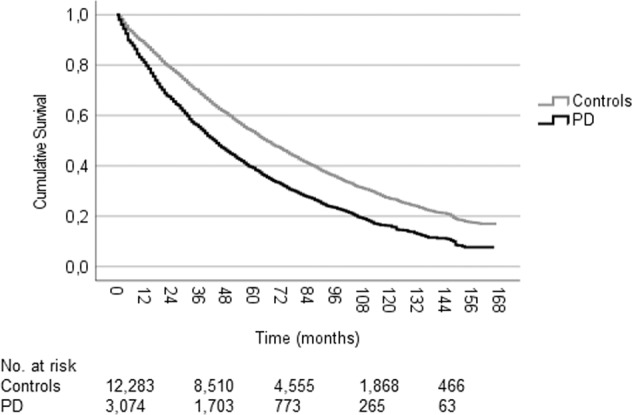
Kaplan–Meier cumulative survival curve for all-cause workforce exit among workforce active persons newly diagnosed with Parkinson’s disease and matched controls. The median survival was 43.0 (95% CI: 40.7–45.3) months in the PD (events: 2341, censored:733) and 66.0 (64.3–67.7) months in the control group (events: 7988, censored: 4295). The median age at diagnosis was 58 years in both groups. Log-rank test *P* < 0.001. PD Parkinson’s disease, CI confidence interval.

The median survival until all-cause workforce exit was 43.0 (95% CI: 40.7–45.3) months among persons with PD that were workforce-active at the time of diagnosis, which was significantly shorter than the 66.0 (95% CI: 64.3–67.7) months in the non-PD control group (*P* < 0.001, Fig. 1).

**Fig. 2:**
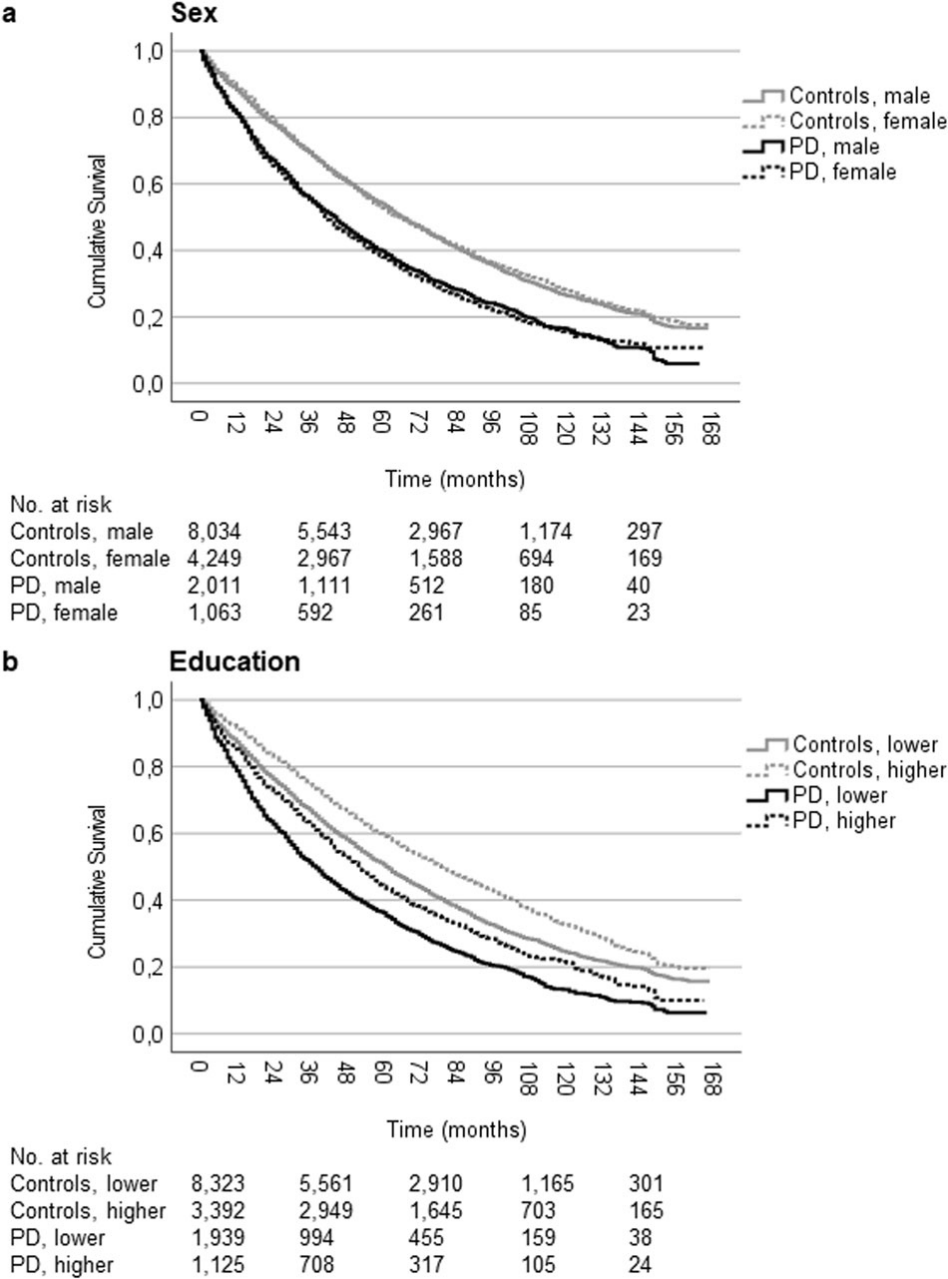
Kaplan–Meier cumulative survival curve for all-cause workforce exit among workforce active persons newly diagnosed with Parkinson’s disease and matched controls by sex and highest attained education. **a** Survival distributions differed significantly between PD cases and controls for both sexes (log-rank tests, both *P* < 0.001), but there were no significant differences between females and males within the PD case or control groups (*P* = 0.545 and 0.433, respectively). The median workforce survival was 42.0 (95% CI: 38.4–45.6) months in female PD patients (events: 816, censored: 247) and 45.0 (95% CI: 41.9–48.1) months in male PD patients (events: 1525, censored: 486). The median survival in the control groups were 66 (95% CI: 63.0–69.0) months in females (events: 2773, censored: 1476) and 66 (95% CI: 64.0–68.0) months in males (events: 5215, censored: 2819). **b** The median workforce survival was 38.0 (95% CI: 35.2–40.8) months in the PD group with lower education (events: 1538, censored: 401) and 52.0 (95% CI: 48.0–55.9) months in the PD group with higher education (events: 803, censored: 322). The survival in the control groups were 62.0 (95% CI: 60.2–63.8) months in the lower (events: 5638, censored: 2685) and 80.0 (95% CI: 76.3–83.7) months in the higher (events: 2350, censored: 1582) education group. Survival distributions tested using log-rank tests, all *P* < 0.001. Lower education: upper secondary school or lower, higher education: post-secondary school or higher. PD Parkinson’s disease, CI confidence interval.

There were no significant differences between sexes in the time to all-cause workforce exit after diagnosis within neither the PD (*P* = 0.545) nor the control (*P* = 0.433) group (Fig. 2a). Workforce-active persons with PD and higher education exhibited a significantly longer median survival until all-cause workforce exit (52.0 [95% CI: 48.0–55.9]) than persons with PD and lower education (38.0 [95% CI: 35.2–40.8], *P* < 0.001) and controls (*P* < 0.001; Fig. 2b).

**Fig. 3:**
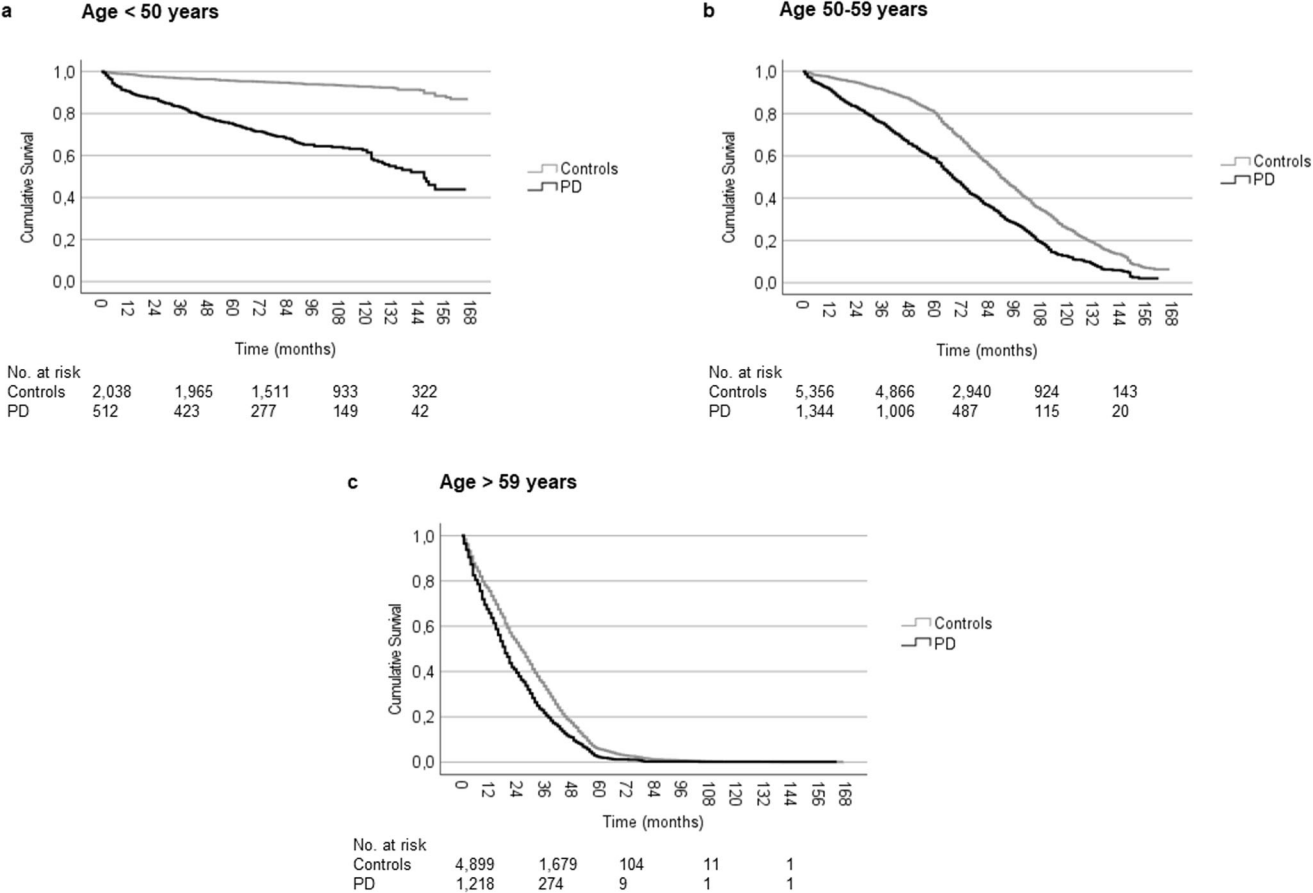
Kaplan–Meier cumulative survival curve for all-cause workforce exit among workforce active persons newly diagnosed with Parkinson’s disease and controls by age at diagnosis/matching. Survival distributions differed significantly between PD cases and controls for each age group (log-rank tests, all *P* < 0.001). **a** The median survival was 147.0 (95% CI: 133.4–160.6) months in PD patients diagnosed <50 years old (events: 188, censored: 324), but could not be estimated in controls <50 years (events: 142, censored: 1896). **b** The median workforce survival was 68.0 (95% CI: 65.1–70.9) months in PD patients 50–59 years (events: 970, censored: 374) and 90.0 (95% CI: 88.4–91.6) months in controls (events: 3171, censored: 2185). **c** The median workforce survival was 19.0 (95% CI: 17.9–20.1) months in PD patients ≥60 years (events: 1183, censored: 35) and 26.0 (95% CI: 25.1–26.9) months in controls (events: 4675, censored: 214). PD Parkinson’s disease, CI confidence interval.

The median survival until all-cause workforce exit was shorter among persons with PD than among controls at all age intervals (all *P* < 0.001; Fig. 3a–c). The median survival until all-cause workforce exit ranged from 147.0 (95% CI: 133.4–160.6) months in workforce active persons diagnosed before age 50 (Fig. 3a) to 19.0 (95% CI: 17.9–20.1) months in workforce active persons diagnosed at age 60 years or older (Fig. 3c).

**Fig. 4:**
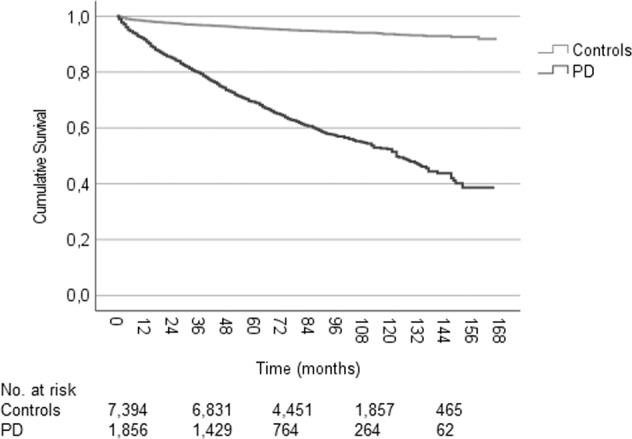
Kaplan–Meier cumulative survival curves for health-related workforce exit among workforce active persons newly diagnosed with Parkinson’s disease and matched controls. Patients and controls younger than 60 years at diagnosis/matching were included. The survival distribution differed significantly between the two groups (log rank test *P* < 0.001). The median time to health-related workforce exit was 123.0 (95% CI: 113.3–132.7) months in the PD group (events: 725, censored: 1131). 7394 controls were included (events = 381, censored = 7013). PD Parkinson’s disease, CI confidence interval.

**Fig. 5:**
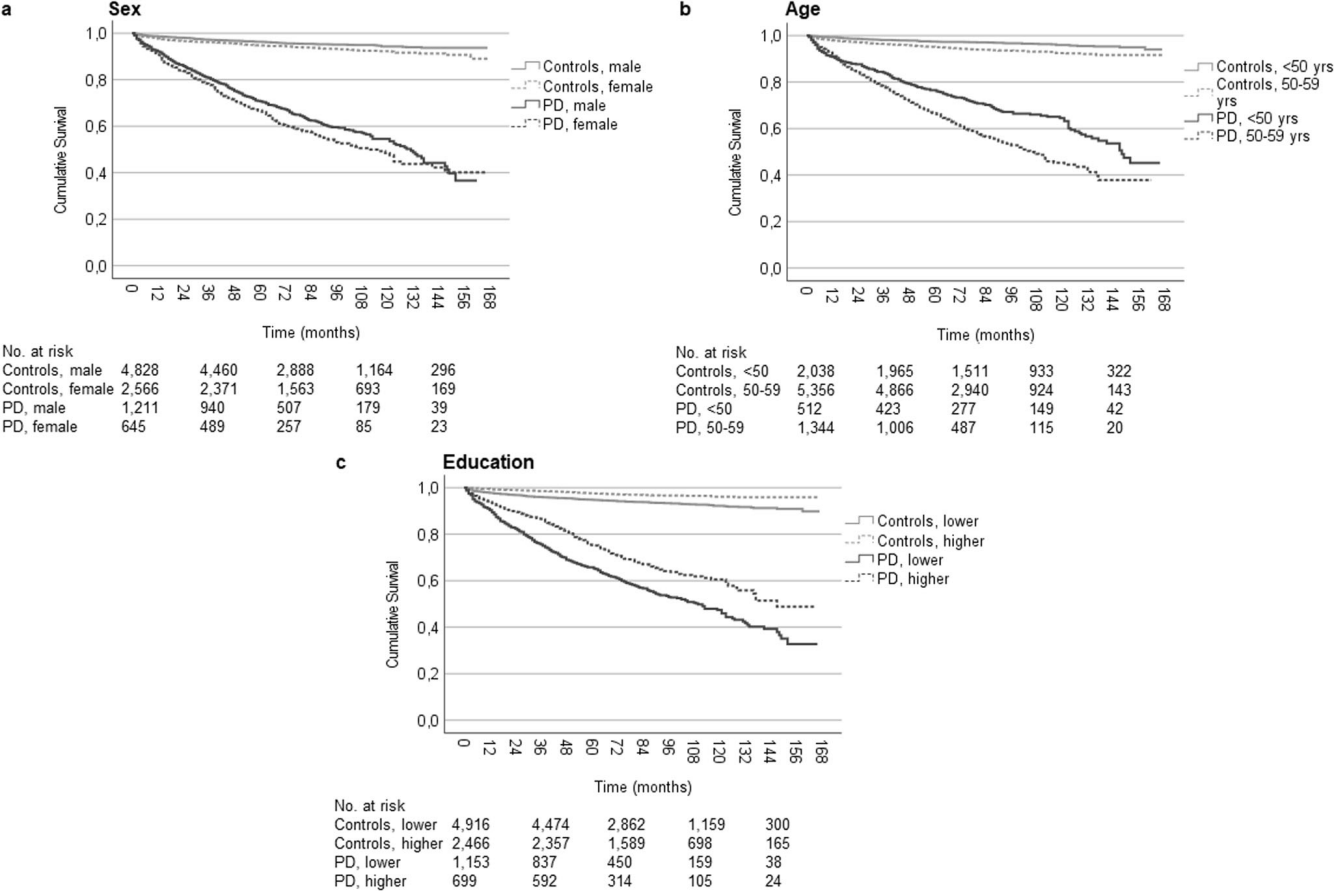
Kaplan–Meier cumulative survival curves for health-related workforce exit among workforce active persons newly diagnosed with Parkinson’s disease and matched controls by sex, age, and highest attained education. Patients and controls younger than 60 years at diagnosis/matching were included. **a** The survival distributions differed significantly between PD cases and controls for both sexes (log-rank tests, both *P* < 0.001), as well as between sexes within the PD case and control groups (*P* = 0.031 and < 0.001, respectively). The median health-related workforce survival was 112.0 (95% CI: 96.1–127.9) months in female PD patients (events: 275, censored: 370) and 131.0 (95% CI: 120.8–141.2) months in male PD patients (events: 450, censored: 761). 7394 controls were included, of whom 4,828 were male (events: 216, censored: 4612) and 2566 female (events: 165, censored: 2401). **b** The survival distributions differed significantly between PD cases and controls for each age group, as well as between both age groups within PD cases and controls (log-rank tests, all *P* < 0.001). The median health-related workforce survival was 107.0 (95% CI: 96.0–118.0) months in PD patients aged 50–59 years at the time of diagnosis (events: 548, censored: 796) and 148.0 (N/A) months in PD patients younger than 50 years at the time of diagnosis (events: 177, censored: 335). 7394 controls were included, of whom 5356 were in the older (events: 307, censored: 5049) and 2038 in the younger (events: 74, censored: 1964) age category. **c** The survival distributions differed significantly between the education groups within PD cases and controls, as well as between PD cases and controls at both levels of education (log-rank tests, all *P* < 0.001). The median time to health-related workforce exit was 110.0 (95% CI: 98.2–121.8) months in the PD group with lower education (events: 498, censored: 655) and 147.0 (N/A) months in the PD group with higher education (events: 227, censored: 472). 7382 controls with lower (events: 306, censored: 4610) or higher education (events: 75, censored: 2,391) were included. Lower education: upper secondary school or lower, higher education: post-secondary school or higher. PD Parkinson’s disease, CI confidence interval, N/A not applicable.

The median survival until health-related workforce exit was significantly shorter in workforce-active persons with PD diagnosed before an age of 60 years (123.0 months [95% CI: 113.3–132.7]) compared to controls (*P* < 0.001; Fig. 4). The median survival until health-related workforce exit differed significantly between men and women with PD (*P* = 0.031; Fig. 5a), as well as between persons diagnosed earlier than an age of 50 years and between ages 50–59 years (*P* < 0.001; Fig. 5b). Furthermore, there were significant differences in the median survival until health-related workforce exit when separated on the level of education in both the PD (*P* < 0.001) and the control group (*P* < 0.001; Fig. 5c).

Using a Cox proportional hazards model, it was found that being 50 years or older at diagnosis (hazard ratio [HR]: 1.56 [95% CI: 1.31–1.85]; *P* < 0.001), having a lower education (HR: 1.54 [95% CI: 1.31–1.80]; *P* < 0.001), and being female (HR: 1.21 [95% CI: 1.05–1.41]; *P* = 0.011) were significant contributing factors to health-related workforce exit in persons with PD (Table 3).

**Table 3:** Cox regression model of health-related workforce exit among workforce active persons (age <60 years) newly diagnosed with Parkinson’s disease.

	*P*	Hazard ratio	95% CI	
Age				
50–59 vs. <50 years	<0.001	1.556	1.309	1.849
Education				
Lower vs. higher	<0.001	1.535	1.312	1.796
Sex				
Female vs. male	0.011	1.214	1.045	1.411

Workforce active persons diagnosed with Parkinson’s disease before an age of 60 years were included (*N* = 1852, events = 725, censored = 1127). The median time to workforce exit or censoring was 63.0 (1st–3rd quartile: 39.0–90.0) months. Health-related workforce exit was defined as two successive years with ≥274 days of sick pay, rehabilitation, or working injury compensation; or one year with ≥274 days of sickness pension. Age: at the time of diagnosis. Lower education: upper secondary school or lower, higher education: post-secondary school or higher.

**Fig. 6:**
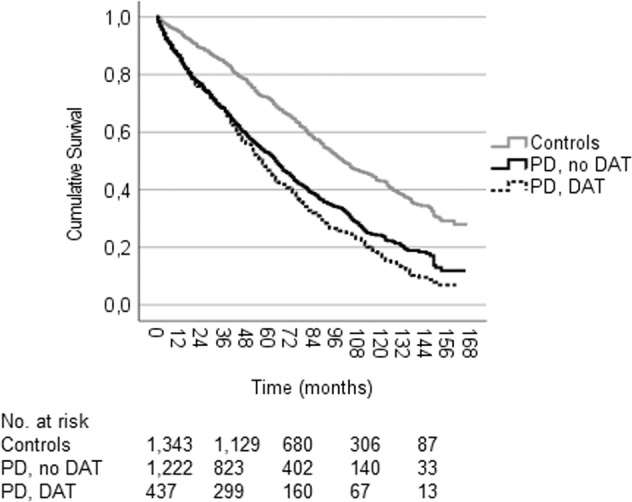
Kaplan–Meier cumulative survival curve for health-related workforce exit after a PD diagnosis in workforce active persons that did or did not receive DAT during follow-up, as well as in non-PD controls. The median workforce survival was 101.0 (95% CI: 93.7–108.3) months in controls (events: 666, censored: 677), 64.0 (95% CI: 59.1–68.9) months in persons with PD and no DAT (events: 794, censored: 428), and 57.0 (95% CI: 51.3–62.7) months in persons with PD and DAT (events: 352, censored: 85). The survival distribution differed significantly in pairwise comparisons between the groups (log-rank tests, *P* < 0.001 except for DAT vs. no DAT: *P* = 0.012). Groups matched for age (±1 year) and sex to PD, DAT. The median age at diagnosis/matching was 55 ± 1 year. In the DAT group, the median time from diagnosis to DAT was 89 (1st–3rd quartile: 66–113.5) months. DAT includes subcutaneous apomorphine infusion, deep brain stimulation, and levodopa-carbidopa intestinal gel. PD Parkinson’s disease, DAT device-aided treatment, CI confidence interval.

There was a significant difference in the workforce survival distribution between persons with PD who did not receive DAT during follow-up compared to persons with PD who did (*P* = 0.012; Fig. 6). The median survival until health-related workforce exit was 64.0 (95% CI: 59.1–68.9) months in persons with no DAT during follow-up and 57 (95% CI: 51.3–62.7) months in persons with initiation of DAT during follow-up. However, persons who received DAT generally did so after they had already left the workforce as the median time from diagnosis to DAT was 89 (1st–3rd quartile: 66–113.5) months.

## Discussion

To our knowledge, this is the largest study on workforce participation in PD to date. The primary finding is that the median workforce survival among workforce active persons receiving a PD diagnosis was about two years shorter than in age- and sex-matched controls (43 months compared to 66 months). Among persons diagnosed with PD during a ten-year period, 33.5% had stopped working at diagnosis, 76.7% within 5 years, and 90.9% within 10 years. We also found that, on the group level, persons that were male, with post-secondary education or higher, and an earlier age of PD diagnosis stayed at work longer.

Previous studies have found the time to loss of employment in workforce active persons with PD to be a median of 7 (95% CI: 4.8–9.2) years^9^, a median of 6 (95% CI: 5.4–6.6) years^8^, or a mean of 4.2 ± 4.4 years^6^. In our study, there were significant differences in median workforce survival depending on the age of diagnosis. For persons diagnosed before an age of 50 years, the median workforce survival was 147 months (Fig. 3A), while it was 19 months for those diagnosed after an age of 60 years (Fig. 3C). Although the prior studies are not dissimilar to our finding of a median survival of 43 months/3.6 years, the age of the study populations and national differences with regards to stipulated retirement age (65 years in Sweden at the time of the study) are likely to influence the time to loss of employment considerably. Nonetheless, for these reasons, comparisons of workforce survival in populations of persons with PD must be made with considerable caution and should preferably include references to matched non-PD populations.

We show that the workforce participation in the PD case group was reduced significantly already at the time of diagnosis in all age groups (Table 2). This is in line with previous studies showing increased prediagnostic morbidity^16,17^, unemployment, healthcare spending^10^, and sick-leave in persons later diagnosed with PD^11^. The established symptoms of prodromal PD—such as rapid eye movement (REM) sleep behavior disorder, autonomic dysfunction, depression, and mild motor signs^18^—can largely explain the increased prediagnostic risk for falls^16,19^, musculoskeletal disorders^11^, constipation, urinary dysfunction, and fatigue seen in previous studies^17^. The National Patient Register does not include primary care visits, but persons in Sweden seeking primary care with a suspected movement disorder are typically referred to a neurologist for diagnosis. We, therefore, think that the considerable majority of newly diagnosed PD patients in the studied age span were included in this study. However, due to the nature of both the healthcare system and the progression of PD, there can be a delay between the first appearances of clinical symptoms to a diagnosis. Together with the potential misclassification of other illness as PD, this may have contributed to the reduced workforce participation already at diagnosis in the PD case group.

We found that higher education is associated with longer workforce survival among persons with PD. Potential explanations for this finding are that education is a proxy for having a higher socioeconomic status and/or cognitive reserve^20^. Persons with higher socioeconomic status generally have more influence over their workday and a stronger connection between effort and reward, which may reduce the risk for job dissatisfaction. Furthermore, both declining health and job dissatisfaction increase the probability for job loss in workers: self-perceived healthy workers who are dissatisfied with their job have a six-fold increase in the risk for health-related job loss^21^. Persons who already rate their health as poor have a higher risk for disability pension, unemployment, and early retirement, while chronic disease and mental health problems increase the risk for disability pension and unemployment, but not early retirement^22^. Education, the type of occupation, and cognitively stimulating leisure activities are markers of a higher cognitive reserve^23,24^, which may help protect against a decline in executive and memory function that can be detrimental for the work ability^20^.

The finding that persons with PD receiving DAT during follow-up had significantly shorter workforce survival than those that did not (Fig. 6) contrasts previous studies indicating that DAT might help sustain working ability^14,15^ and performance of daily activities^13^ in PD. However, DATs are typically initiated during mid-to-advanced stages of PD, at which point a significant portion of working-age persons with PD have already left the workforce^1^. Our finding that the median time from diagnosis to initiation of DAT exceeds the median workforce survival in the DAT group (89 versus 57 months) underscores this problem.

The large number of workforce active persons with PD included is a unique strength of this study. However, the study has several limitations that need to be considered in the interpretation of the results. Firstly, the percentage of misclassified PD in the National Inpatient Register is not known, but it is likely that the case group included a minority of persons with atypical parkinsonism or other movement disorders. This is also supported by the surprisingly large proportion of deaths during follow-up in the case group, which could be the result of a misclassification of persons with more rapidly progressing movement disorders than PD. The overall positive predictive value of the Inpatient Register has been shown to be between 85–95% depending on the diagnosis^25^. Secondly, any misclassification of younger persons is likely to result in larger discrepancies as the expected workforce survival is long. Furthermore, the data we had access to lack information on many of the disease-specific characteristics that are likely to influence workforce survival in PD. This information could for example have improved the accuracy of the matching between persons with PD and either DAT or not.

Persons with young- or early-onset PD are typically progressing at a slower rate than those with later onset and have been shown to exhibit distinct biochemical features^26–28^. Although the time to loss of employment is longer in persons with earlier PD onset, so is the time of lost employment and the amount of lifetime earnings loss^29^. The slower disease progression and more future workforce participation and income at risk make persons with early-to-mid PD onset ideal for future interventions aiming to improve workforce participation after a PD diagnosis. However, the increased probability for underlying predisposing mutations in younger persons with PD must be considered as this may affect the generalizability of findings.

This study was based on the total Swedish population and just as for most previous studies on workforce participation in PD, the unique setup of a single country’s healthcare and social security system may affect the generalizability of the findings. Nevertheless, some of the findings are of particular interest. Workforce participation is reduced already at the time of a PD diagnosis and continues to decline during the course of the disease. The effect of DAT on working ability in PD is still not clear and, according to current routines, DATs are often introduced at a stage in the disease when many have already stopped working. Studies on the effect of DAT on the work ability of workforce active persons with PD are warranted. Supportive measures intended to facilitate workforce participation are needed shortly after the PD diagnosis has been established and structured interventions aiming to improve workforce participation for this population are warranted.

## Methods

### Study design

This was a nested case cohort study. The base population used for the study was the total Swedish population (which reached 10 million in January 2017). Cases were identified from the Swedish National Patient Register and controls from the Total Population Register at Statistics Sweden. Individual-level endpoint and exposure data were collected from additional national registers. The primary endpoint measure was all-cause workforce exit. Health-related workforce exit was used as secondary outcome measure.

### Data sources

Data were retrieved from registers held by the Swedish National Board of Health and Welfare and Statistics Sweden. Data from the National Patient Register, the National Prescribed Drug Register, the Cause of Death Register, and the Longitudinal Integrated Database for Health Insurance and Labor Market Studies (LISA) were merged into a dedicated study database for the analyses.

The National Patient Register contains patient, geographical, administrative, and medical data from inpatient (since the 1960’s) and outpatient (since 2001) specialist healthcare provided by both private and public caregivers in Sweden. Primary care visits are not included in the register. Individual-level data on the first occurrence of a PD diagnosis (for cases) and deep-brain stimulation surgery were added to the study database for both cases and controls.

The National Prescribed Drug Register contains patient, product, and prescription data, costs, and prescriber information on all prescribed drugs dispensed at pharmacies in Sweden. The register was established in July 2005. The date of first prescription of drugs commonly used in the treatment of PD was added to the study database for both cases and controls.

Established in 1961, the Cause of Death Register is a compilation of the cause-of-death certificates that must be sent to the National Board of Health and Welfare within three weeks following the death of a person in Sweden. The certificate states the cause of death and co-morbidities or circumstances contributing to the death according to the International Statistical Classification of Diseases and Related Problems (ICD-10) system. The date of death was added to the study database.

LISA was established in 1990 and contains information on demographics, education and training, employment and unemployment, income, and social insurance. Persons 15 years or older that were registered in the Swedish population on December 31st of each year are included. LISA was the basis for sociodemographic and workforce-related information for cases and controls in the study database.

### Study populations

From the base population, Swedish citizens aged 18–64 years residing in Sweden and receiving a first PD diagnosis in the National Patient Register during the years 2003–2012 were identified. Persons with a registered PD diagnosis during the years 2001–2002 and persons who immigrated to Sweden during 2001–2016 were excluded to increase the certainty of the date of diagnosis. The identified cases were initially matched in a 1:5 ratio to controls from the Swedish population using Statistics Sweden’s Total Population Register using the year of birth, sex, and municipality of residence at the time of diagnosis to form a primary case-control study population. Taking also workforce status at time of diagnosis into account, a second matching at a 1:4 ratio was made to form a secondary case-control study population for the survival analyses. In a third matching, non-PD controls, persons with PD and no DAT during follow-up, and persons with PD and DAT during follow-up were matched on age at diagnosis/matching (±1 year) and sex. Cases were not allowed to be used as controls prior to becoming a case and controls were allowed to be drawn only once. The last day of inclusion was December 31st 2012 and the end of follow-up was December 31st 2016.

### Statistical analyses

The primary study population was presented using descriptive statistics. Differences between the PD cases and controls regarding age, sex, place of birth, medication, DAT during follow-up, workforce participation at diagnosis, death during follow-up, and emigration during follow-up were tested using *χ*^2^ or Mann–Whitney *U* tests depending on the type of data.

Individuals in the study populations were followed from the date of recording until workforce exit, death, emigration, or end of follow-up. Analyses of determinants of workforce exit were performed with workforce active cases matched at the time of inclusion to workforce active controls on age and sex. The Kaplan–Meier method was used to investigate the time to all-cause and health-related workforce exit among persons with PD and controls. All-cause workforce exit was defined as the first occurrence of either death, two successive years without information from employer on income, one year with ≥274 days of sickness pension, one year with full pension; two successive years with ≥274 days of sick pay, rehabilitation, or working injury compensation; or two successive years with ≥274 days of unemployment. Health-related workforce exit was defined as two successive years with ≥274 days of sick pay, rehabilitation, or working injury compensation; or one year with ≥274 days of sickness pension. The median survival was defined as the length of time from the point of diagnosis or matching to the point at which half of the persons originally at risk had reached the event. Log-rank tests were used to investigate differences in survival distribution.

A Cox proportional-hazards model was used to investigate associations between workforce survival time and age, sex, and educational level.

IBM SPSS version 27.0 was used for statistical analyses. *P* < 0.05 was considered statistically significant. Data were stored, handled, and analyzed according to the General Data Protection Regulation (GDPR) on the Lund University secure data management platform LUSEC.

## Data Availability

No additional data can be provided by the authors. Data from the named databases are available through request from the respective government agencies.

## Code availability

The underlying code for this study is not publicly available but may be made available to qualified researchers on reasonable request to the corresponding author.
